# Short Term Interactions with Long Term Consequences: Modulation of Chimeric Vessels by Neural Progenitors

**DOI:** 10.1371/journal.pone.0053208

**Published:** 2012-12-27

**Authors:** Cicely Williams, Millicent Ford Rauch, Michael Michaud, Rebecca Robinson, Hao Xu, Joseph Madri, Erin Lavik

**Affiliations:** 1 Interdepartmental Neuroscience Program, Yale University, New Haven, Connecticut, United States of America; 2 Department of Biomedical Engineering, Yale University, New Haven, Connecticut, United States of America; 3 Department of Pathology, Yale University, New Haven, Connecticut, United States of America; 4 Department of Biomedical Engineering, Washington University, St. Louis, Missouri, United States of America; 5 Department of Biomedical Engineering, Case Western Reserve University, Cleveland, Ohio, United States of America; Johns Hopkins University, United States of America

## Abstract

Vessels are a critical and necessary component of most tissues, and there has been substantial research investigating vessel formation and stabilization. Several groups have investigated coculturing endothelial cells with a second cell type to promote formation and stabilization of vessels. Some have noted that long-term vessels derived from implanted cocultures are often chimeric consisting of both host and donor cells. The questions arise as to whether the coculture cell might impact the chimeric nature of the microvessels and can modulate the density of donor cells over time. If long-term engineered microvessels are primarily of host origin, any impairment of the host's angiogenic ability has significant implications for the long-term success of the implant. If one can modulate the host versus donor response, one may be able to overcome a host's angiogenic impairment. Furthermore, if one can modulate the donor contribution, one may be able to engineer microvascular networks to deliver molecules a patient lacks systemically for long times. To investigate the impact of the cocultured cell on the host versus donor contributions of endothelial cells in engineered microvascular networks, we varied the ratio of the neural progenitors to endothelial cells in subcutaneously implanted poly(ethylene glycol)/poly-L-lysine hydrogels. We found that the coculture of neural progenitors with endothelial cells led to the formation of chimeric host-donor vessels, and the ratio of neural progenitors has a significant impact on the long term residence of donor endothelial cells in engineered microvascular networks in vivo even though the neural progenitors are only present transiently in the system. We attribute this to the short term paracrine signaling between the two cell types. This suggests that one can modulate the host versus donor contributions using short-term paracrine signaling which has broad implications for the application of engineered microvascular networks and cellular therapy more broadly.

## Introduction

Many of the tissues we seek to engineer are highly vascularized with the vasculature being critical to the tissue function and survival. It is not surprising, then, that there has been significant research focusing on methods to promote rapid vascularization of engineered tissues. There are a number of methods to promote vascularization including incorporation of growth factors to stimulate angiogenesis from the host [1], [2], prevascularization of the engineered construct in vivo in regions such at the omentum [3], [4], and seeding scaffolds with cells to preform vascular networks which can be implanted in vivo and rapidly inosculate with the host [5].

In some of the early work looking at seeding cells to preform vascular structures, the focus was on using endothelial cells of different origins [6]–[8]. It is a logical choice: endothelial cells line vessels and in the microvasculature they are the predominant cell type. Over time, they become invested by pericytes. The pericyte investment has been shown to be critical to the function of microvessels. Implantation of endothelial cell-seeded scaffolds often lacks pericytes investment at long times, and vessel regression is seen [9]–[12]. These observations motivated the study of the role of coculture of endothelial cells with other cell types which either could invest the microvessels or provide paracrine signaling to the host pericytes to invest the microvessels in implants.

The coculture of endothelial cells with a range of cells including osteoblasts [13], mesenchymal stem cells [14]–[17], and neural progenitors [18]–[20] has been shown to impact the formation and stabilization of vessels.

A number of groups have reported chimeric vessels consisting of some donor ECs in long term implants [21]–[25]. The questions that arise now that one can engineer stable microvessels are what role does the coculture cell have on the host versus donor contributions to the microvascular networks over time, and how might this contribution be modulated.

These questions are important for several reasons. The first reason is that endothelial cells have the potential to lead to an immune response. If the donor endothelial cells are not present for long times, one may have very different approaches to dealing with the immune response than if they are present indefinitely. The second reason is that engineering microvascular networks becomes a powerful tool to deliver molecules a patient lacks systemically for long times via genetically engineering the donor endothelial cells [26]. For example, certain hemophiliacs lack factor VIII. Endothelial cells have been transduced or transfected to secrete factor VIII for hemophilia [27]–[30]. However, for this approach to work, the cells must incorporate in the host vasculature and remain present for long times. Other examples of the secretion of molecules from endothelial cells include the delivery of human growth hormone [31], and molecules to induce apoptosis in tumors [32]–[34]. The third reason is that if long term, engineered microvessels are primarily of host origin, any impairment of the host's angiogenic ability has huge implications on the long term success of a tissue engineered structure. If one can modulate the host versus donor response, one may be able to overcome impairment of the host.

We sought to investigate the impact of the coculture cell on the host versus donor composition of engineered microvessels over time. Our lab has been looking at the impact of cocultures of neural progenitors with endothelial cells in engineered microvascular networks for some time. Neural progenitors produce factors that influence endothelial cells and microvessels [18]–[20], [35] and provide a means to investigate modulating vessel behavior. Therefore, in this work, we have varied the ratio of the neural progenitors to endothelial cells to investigate the nature of engineered vessels over time and to determine whether the host versus donor contributions to vessels can be influenced by the neural progenitors. We found that the coculture of neural progenitors with endothelial cells led to the formation of chimeric host-donor vessels, and the ratio of neural progenitors has a significant impact on the long term residence of donor endothelial cells in engineered microvascular networks in vivo even though the neural progenitors are only present transiently in the system. We attribute this to the short term paracrine signaling between the two cell types. This suggests that in engineered microvascular structures, we can modulate the host versus donor contributions which had broad implications for the application of tissue engineered microvascular networks as well as the understanding of cell behavior post transplantation more broadly.

## Methods

### Macroporous hydrogel synthesis

Macroporous hydrogels were synthesized as described in [18]. Briefly, four-arm PEG (MW ∼10,000 g/mol) was activated and added to PLL (MW ∼70–110 kDa) in deionized water for a final ratio of 1∶4 PEG hydroxyls to PLL free amines. The polymer solution was cast over a salt-leached poly(lactic-co-glycolic acid) (PLGA) (Resomer 506) scaffold with 250–500 µm pores [36] and allowed to cure at room temperature for 24–36 hours. The PLGA scaffold was then removed by degradation in 3 M sodium hydroxide to completion.

### Cell Culture

Immortalized BECs, a generous gift from Britta Engelhardt (Theodor Kocher Institute, Bern, Switzerland) [37], [38] were maintained in BEC medium (DMEM, 10%FBS, 10 mM HEPES, 1% Antibiotic-Antimycotic). Green fluorescent protein (GFP) NPCs were isolated and maintained according to Lu et al [39] in serum-free DMEM/F12 containing L-glutamine, N2, B27, and EGF. Briefly, SVZs were dissected from GFP+ postnatal day 0 to day 3 mouse brains. Tissue was partially digested in 0.1% collagenase and filtered through a 70 µm cell strainer. Cells were resuspended in NPC media and monitored for the formation of neurosphere aggregates.

*Hydrogel Seeding*. Hydrogel discs 5 mm in diameter and 1 mm thick were cut from larger hydrogels. After UV sterilization cells were seeded on the hydrogel discs at the following ratios. For the low ratio coculture, gels were seeded at a ratio of 1∶10 (100,000 NPCs∶1 million BECs). For the high ratio coculture treatment group, gels were seeded at a ratio of 1∶1 (1 million NPCs∶1 million BECs) on day one and an additional 1 million NPCs were added on day three for a final ratio of 2∶1. High ratio coculture NPCs were also lightly triturated to create smaller secondary neurospheres that could better integrate into the BEC network. Other studies have demonstrated that secondary neurospheres are morphologically and phenotypically identical to their primary counterparts and possess the same potential for differentiation [40]–[42]. Seeded gels were maintained in BEC media under static conditions in 24-well plates for 3 days at 37°C and 5% CO_2_. This seeding protocol resulted in final NPC∶BEC ratios of 1∶10 for the low ratio coculture and 2∶1 for the high ratio coculture.

### Surgical Procedures

Eight- to ten-week-old female C57 black mice (C57BL/6, Charles River Laboratories, Wilmington, MA) were anesthetized with an i.p. injection of ketamine (100 mg/kg) and xylazine (20 mg/kg). Two separate 1 cm long incisions were made through the skin overlying the upper thoracic and upper lumbar vertebrae. Subcutaneous pockets were created bilaterally by clearing connective tissue under the skin. Two to four hydrogels from the same treatment group were implanted in each mouse, one in each pocket. The incision was closed with surgical clips, and the animals were maintained on a heating pad until they regained mobility. All procedures were approved by the Animal Care and Use Committees of Yale University. At nine and twelve weeks, animals were injected retro-orbitally with 100 µL of biotinylated tomato lectin at 1 mg/mL (Vector Laboratories). Lectin was allowed to circulate for 5–10 minutes prior to implant retrieval.

### Histology and Immunocytochemistry

*In vitro* gels and excised implants were fixed in 4% paraformaldehyde for 1–2 h, soaked in a 30% sucrose solution overnight, and embedded in OCT. Three representative implants per study group were randomly chosen (each from a different animal) for sectioning. 20-µm-thick cross sections were cryosectioned. Sections were stained with hematoxylin and eosin (H&E) or immunostained with antibodies against mouse platelet endothelial adhesion molecule-1 (PECAM-1) (Pinter et al [43]., 1∶100), and NG2 proteoglycan (Chemicon; 1∶200), and polyoma middle T antigen (Pymt) (Calbiochem; 1∶200). Secondary antibodies included goat anti-rabbit, goat anti-mouse Alexa Fluor 647 (Molecular Probes; 1∶200), and streptavidin Texas-RedX (Molecular Probes; 1∶150). Briefly, 20-µm sections were blocked in 5% BSA and 3% normal goat serum (Vector Laboratories) for 1 h at room temperature. Samples were then incubated with the primary antibody at 4°C overnight followed by incubation with the appropriate secondary antibody for 1 h at room temperature. At least five sections, 200 µm apart, were stained for trichrome, H&E, and each immunocytochemical marker.

### Quantification of Histology

The total number of microvessels and the number of microvessels containing graft ECs were quantified using a 20× objective on the Zeiss Axiovert 200 M microscope with an MRm camera and Axiovision 4.5 software for image capture and analysis. Vessels were identified by their immunostaining for PECAM-1 and their morphology. For each quantification, a total of ten areas were counted in each of the three sections per implant to obtain representative samples for statistical analysis.

### ELISAs

To test the expression of VEGF, BDNF, PDGF-BB, Ang-1, and Ang-2 in *in vitro* hydrogel cultures, ELISA analysis was carried out on cell culture medium from three independent experiments following the manufacturer instructions for the appropriate ELISA kit (Quantikine; R&D Systems). For each independent experiment, al least twelve hydrogels were combined for analysis.

### Statistics

All experiments were done in triplicate and data analyzed using a two-way ANOVA followed by the Tukey test to determine differences between groups. The statistical significance threshold was p<0.05.

## Results

### Morphology and density of vessels in vitro and in vivo

#### In vitro implant morphology and cord density

We examined two ratios of NPCs to BECs in this work, a low ratio (1∶10 NPCs∶BECs) and a high ratio (2∶1 NPCs∶BECs) coculture. The low ratio culture mimics our previous work building microvascular networks in macroporous PEG/PLL hydrogels [18], [20]. We hypothesized that increasing the ratio of NPCs would impact the residence of the BECs over time. In our previous work, we had seen that the ratio of NPCs could impact tube formation in two dimensions, so we investigated the impact of the NPC ratio on tube formation in the hydrogels.

**Figure 1:**
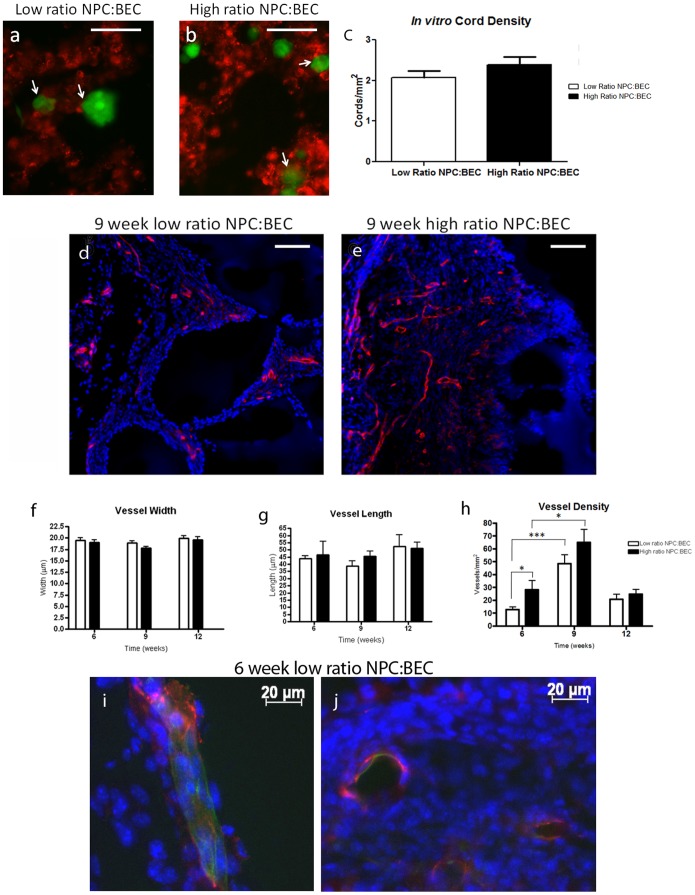
In vitro and in vivo vessel morphology and density. (a–b) Live imaging of the low ratio (a) (1∶10 NPCs∶BECs) coculture and high ratio (2∶1 NPCs∶BECs) on day 3 in the PEG/PLL macroporous hydrogel. The NPCs are GFP-positive (green) and the BECs are labeled with DiI (red). Scale bar 100 um. (c) Quantification of the in vitro BEC cord density showing no differences in cord density at 3 days post seeding in the macroporous gels. (d) Representative images of PECAM-1 immunostaining (red) and DAPI counterstaining from the core of the low ratio (d) and high ratio (e) coculture implants at 9 weeks post implantation. Scale bar = 100 um. Quantification of the vessel width (f) and vessel length (g) in the two groups showed no differences over time. Vessel density (h) showed a small difference between the groups at 6 weeks (p<0.05) but no differences between the groups at 9 or 12 weeks. Vessel density increased transiently at 9 weeks for both groups (p<0.05) transiently with vessel density returning to the 6 week values at 12 weeks post implantation. Pericytes were identified by immunostaining for NG2 (green) and found to be investing the vessels. Representative images from 6 weeks post implantation (i–j).

We imaged the gels on day three, the day at which tube formation is visible [18]. Live cell imaging showed that in both groups, NPCs were in contact with BEC cords (Fig. 1a–b, arrows), which mimics the organization seen in the neurovascular niche and in other progenitor cell vascular niches [44]–[46]. However, the high ratio NPC∶BEC coculture had a greater number of NPCs which were more evenly dispersed into the BEC network than the low ratio coculture as one would expect.

We quantified the number of endothelial cords per mm^2^ by cryosectioning the implants and immunostaining for platelet endothelial cell adhesion molecule (PECAM-1) an EC cell marker [47]. Both groups had the same number of cords per mm^2^ independent of the ratio of NPCs (Fig. 1c). BECs lined the hydrogel pores, and the density of macropores appears to dictate the density of cords in these *in vitro* cultured hydrogels.

### In vivo vessel morphology and quantification

5 days after seeding, the macroporous hydrogels were implanted subcutaneously in C57BL/6 mice. The implants were harvested 6, 9, and 12 weeks post implantation, cryosections, and immunostained for PECAM-1 (Fig. 1d, e). We chose to look at these long time points because the effects of angiogenesis associated with wound healing are avoided [48], [49] and because the concentration of donor endothelial cells typically drops precipitously at these times [50], [51]. In both coculture groups, PECAM-positive microvessels were seen throughout the implants at these long time points. This is in marked difference to EC-alone implants which we had seen previously show significant vascular regression by 6 weeks post implantation [18].

Quantification of vessel length and diameter showed no differences between the groups over any of the time points observed. The vessel diameter in both groups was approximately 20 um. There were larger feeder vessels (30–50 um) at the very periphery of the implants, but no vessels of that size were found within the bulk of the implants for either group (Fig. 1f, g). The ratio of NPCs does not impact the morphology of the vessels. The ratio, however, does modestly impact the density of vessels at 6 weeks but not at longer time points (Fig. 1h). At 12 weeks post implantation, the density of vessels decreases in both groups compared to the 9 week data to the same levels seen at 6 weeks. Microvessels are still found throughout the entirety of the implants with no signs of central vessel regression.

For microvessels to be stable over long times, they must become invested with pericytes [12], [52]. We stained immunostained the implants for NG2, a proteoglycan expressed by pericytes [53]. Both the low ratio and high ratio groups showed investment of PECAM-positive vessels with NG2-positive pericytes (Fig. 1i, j) which helps to explain the long term stability of these microvessels. There were no obvious differences in the investment of pericytes in either group.

### NPC Behavior over time

**Figure 2:**
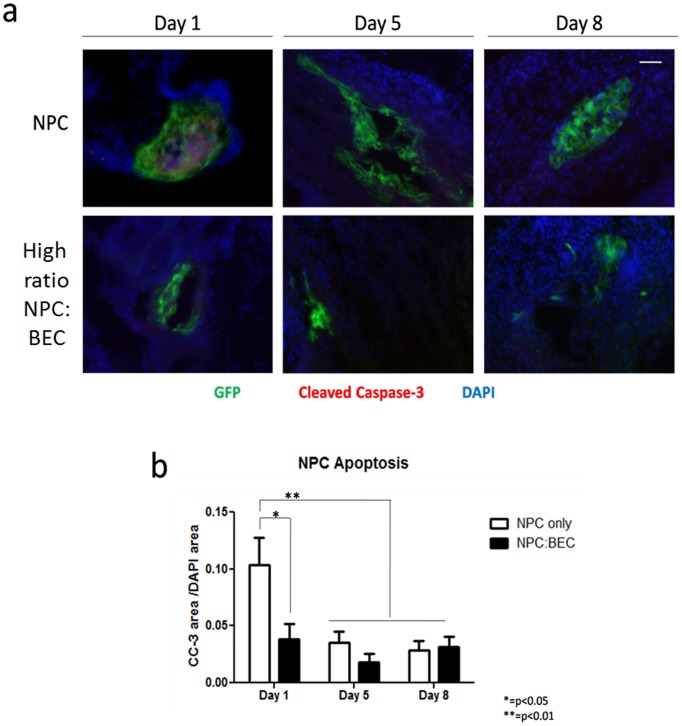
GFP+ NPC Implant Apoptosis. Implants were sectioned and immunostained for the early effector caspase, cleaved caspase-3 (red, GFP = green, DAPI = blue) and area of fluorescence relative to DAPI was quantified (b). On day one, the rate of implanted NPC apoptosis was significantly decreased when NPCs were co-implanted with BECs (0.103±0.025 versus 0.039±0.014, p = 0.0475). This initially higher rate of apoptosis significantly decreased by day 5 (0.036±0.050, p = 0.0063) to a rate comparable to that of the NPC∶BEC implants. This rate did not change at day 8 (0.029±0.008). The rate of NPC apoptosis in the NPC∶BEC implant, remained the same over time (day 1: 0.039±0.014, day 5: 0.018±0.008, day 8: 0.032±0.009). Error bars correspond to ± SE. Scale bar: 50 µm.

In all of the sections observed, no NPCs were detected at these long time points. This compliments our previous work, where we found that mouse NPCs were resident for less than a week in these coculture implants [18] and only slightly more than a week in the rat NPC implants [20]. In both cases, the short term residence of the NPCs had long term implications for the stability of microvascular networks in the implants. We did do a series of short term experiments over the first 7 days post implantation to track the NPCs, and we found that the number decreases rapidly over that time. We stained the sections with cleaved caspase-3 to look for signs of apoptosis, and the NPCs, which rapidly moved to the periphery, were not positive (Fig. 2a). Quantification of NPC apoptosis over time demonstrates that beyond the first day, there is very limited apoptosis (Fig. 2b). Quantification of apoptosis at day 1 demonstrates that the coculture of BECs with NPCs reduces NPC apoptosis at this short time point which is consistent with our previous findings [19].

**Figure 3:**
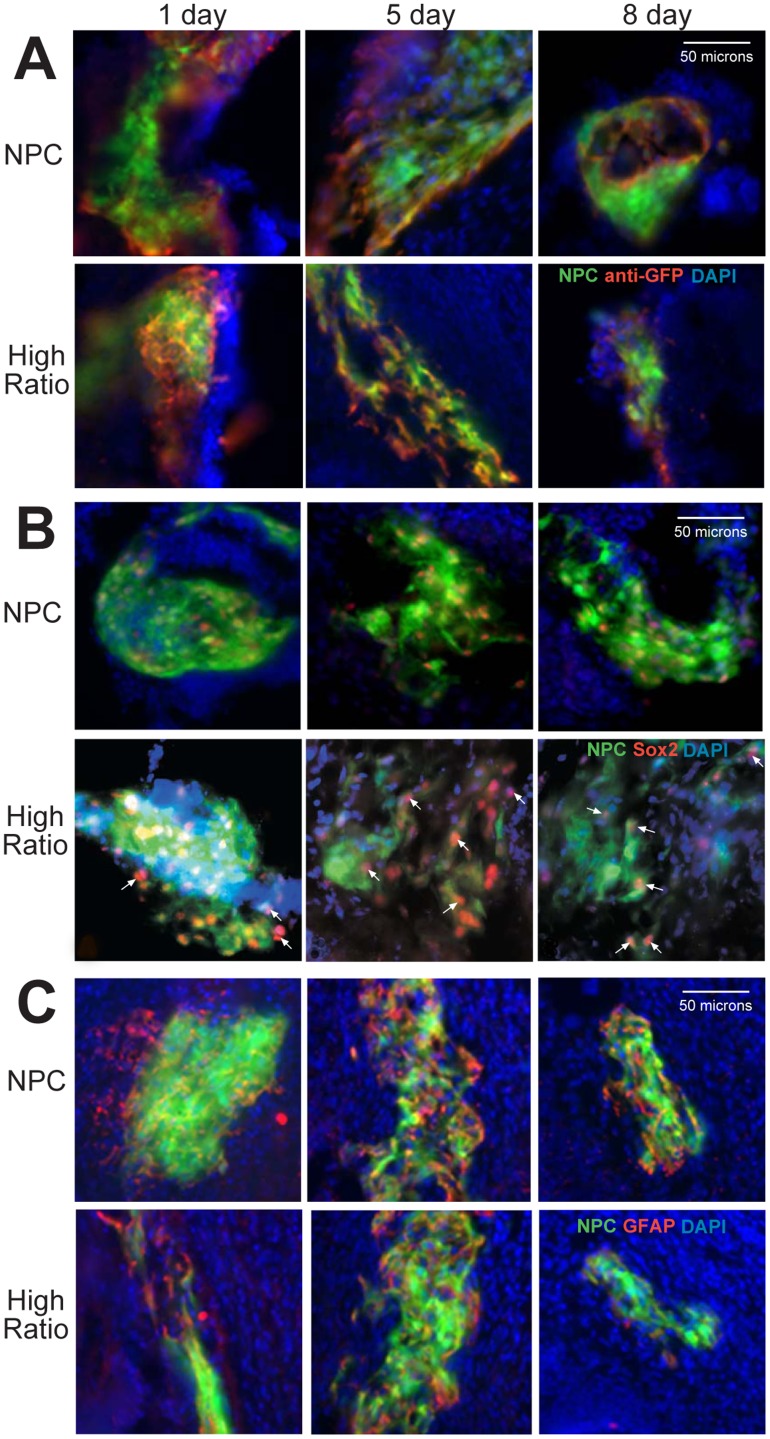
GFP+ NPC Implant GFP and Differentiation Marker Expression. Implants were sectioned and immunostained with an antibody to GFP (a, red) to assess GFP downregulation (GFP = green, DAPI = blue). In both NPC only and high ratio NPC∶BEC groups, at all time points central clusters of GFP+ cells were surrounded by a thin border of cells that did not exhibit green fluorescence but which became visible upon staining (a). Sections were also immunostained with sox 2(b, red) to assess differentiation status of the GFP+ NPCs. Sox2 staining remained in the nuclei of GFP+ cells only over time and across treatment groups (b). Finally, sections were immunostained with GFAP to determine their differentiation status (c, red). GFAP staining overlapped with GFP+ staining and also stained a thin surrounding border of GFP- cells just beyond the central GFP+ cluster (c). Scale bars: 50 µm.

Since apoptosis of NPCs was not substantial enough to fully account for their decreased presence in implants over relatively short time points, we explored the possibility that some NPCs remained in the implant, but were not observed due to a downregulation of GFP. The transgenic animals used in this study expressed GFP driven by a chicken β-actin promoter with a cytomegalovirus (CMV) enhancer that is typically more resistant to differentiation-induced downregulation than either transcriptional element alone [54]. However, these studies were done with in vitro-induced differentiation in embryonic stem cells. To assess GFP downregulation in our system, GFP immunostaining was performed. At all time points tested, central clusters of GFP+ NPCs were surrounded by an extension of cells which did not exhibit green fluorescence but which became visible when stained with an antibody to GFP (Fig. 3a). This external border of cells did not expand over time and remained just proximal to the GFP+ central NPC cluster. Even using the GFP antibody, we did not find cells in the implant beyond 8 days post implantation.

Another important post-implantation consideration is the differentiation of NPCs within the host tissue. To assess differentiation status, implants were stained with sex determining region Y-box 2 (sox 2), which is a transcription factor that maintains self-renewal in undifferentiated cells [55] or glial fibrillary acidic protein (GFAP), which labels both immature progenitor cells and mature glia. Over time, the central GFP+ cells maintained sox 2 staining with no differences seen due to treatment group or time (Fig. 3b). Notably, no sox 2 staining was seen just outside of the central GFP+ cluster where cells downregulating GFP are located. GFAP expression was found in external border cells in the same relative location as those cells that were stained with anti-GFP (Fig. 3c). Similarly, an extension of this border was not seen over time. Cells were also stained with the neuronal marker β-III tubulin, but no positive staining was seen in either group at any time point.

Based on all of these findings, we hypothesize that the cells are migrating out of the implant, although we do not know at this time, where they may be migrating to.

### Vessels actively remodeling even at 12 weeks post implantation

**Figure 4:**
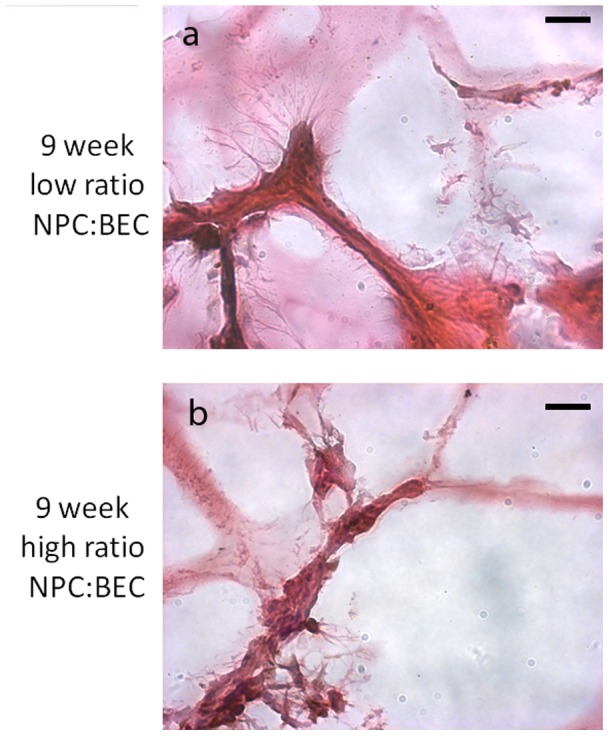
Hematoxylin and eosin (H&E) staining revealed that even at 12 weeks post implantation there are angiogenic tip cell present in both the low (a) and high (b) ratio groups, and there is active vessel remodeling occurring. Scale bar = 25 um.

One of the important things to remember with microvascular implants is that they are dynamic systems capable of extensive remodeling over time in response to the environment. Even at 12 weeks post implantation, we find angiogenic endothelial tip cells [56], [57] actively investigating the environment (Fig. 4). Even though we have found the vessels to be functional, stable, and invested with pericytes, the vessels continue to be dynamic and investigating their environment as evidenced by tip sprouting.

### Modulation of chimeric vessels by NSCs

#### Anastamosis of vessels with host

We investigated anastamosis using tomato lectin. Prior to implant excision, we injected biotinylated tomato lectin into the host vasculature via retro-orbital injections to label implant vessels which had anastamosed with the host. Tomato lectin binds to specific disaccharide moieties of glycosaminoglycan chains which are found in the EC glycocalyx making it an effective marker of vasculature. The intravenous route of administration restricts the vessels labeled to those that have anastamosed with the host vasculature.

We found tomato lectin lining vessels throughout the implants including in the central region at all time points suggesting that the vessels are anastamosed. This is not surprising given the time course we studied in this work. There were no differences in the nature or density of tomato-lectin labeled vessels between the low and high ratio groups.

#### Host versus donor contributions of endothelial cells to vessels

The key question in this work was whether we could modulate the host versus donor contributions to the microvasculature over time. Varying the ratio of the NPCs did not have a significant effect on the vessel morphology, density, or functionality, but we did see that it had a significant effect on the donor cell contribution.

**Figure 5:**
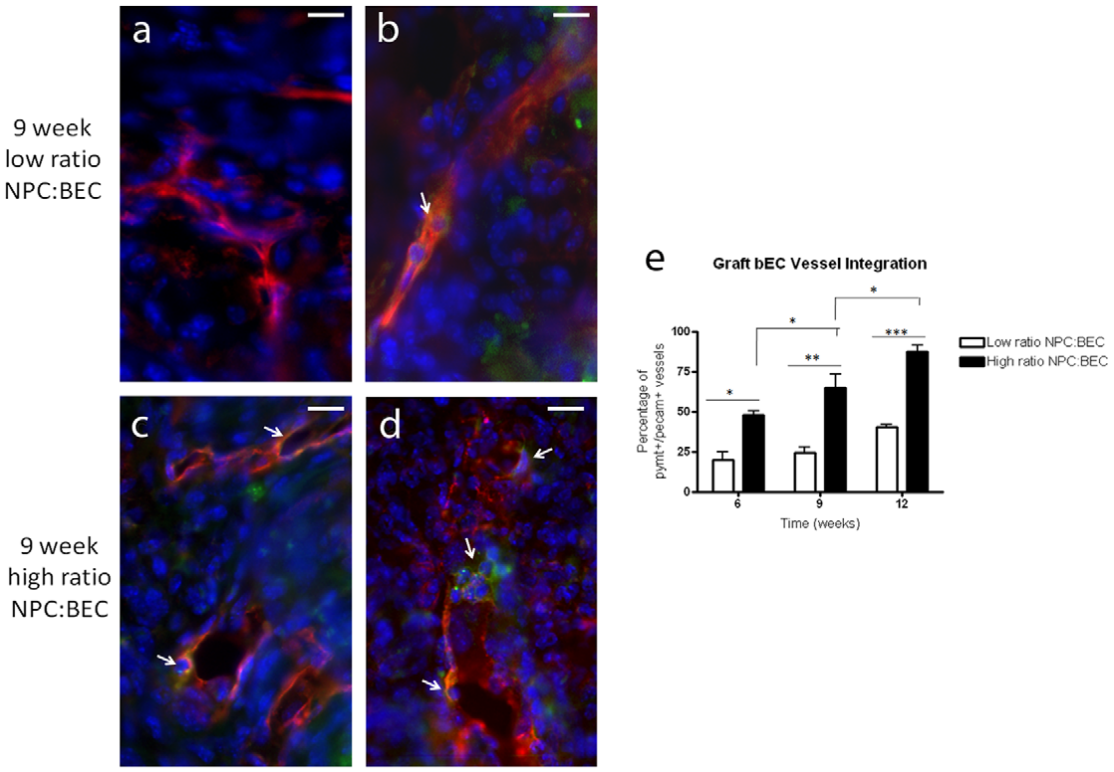
Donor versus host contributions to vessels over time. Donor BECs were immunostained with an antibody to PymT (green) and PECAM-1 (red) with DAPI for nuclei in the low ratio (a,b) and high ratio (c,d) groups. Quantification (e) showed that the ratio of NPCs modulates the percentage of vessels with donor (PymT, green) cells at all time points.

The donor endothelial cells used in this work were immortalized by the insertion of a gene for the polyoma middle T antigen (PymT). PymT promotes the expansion of endothelial cells [58], which is critical when working with mouse endothelial cells which do not expand readily. We used an antibody to PymT to identify the donor cells. Implants were sectioned and co-stained with PECAM-1 and PymT to visualize vessels containing graft BECs (Fig. 5 b–c, e–f). Chimeric vessels were present which contained PymT −/PECAM-1+ host cells and PymT +/PECAM-1+ graft cells. Notably, in these chimeric vessels, multiple graft cells were seen per vessel and they were evenly distributed along the length of the vessels with no clear demarcation visible between host and graft contributions (Fig. 5 a–b, c–d, arrows).

We saw no clear differences in the number of donor cells per vessel in the low and high ratio cocultures. We quantified the number of vessels with donor cells over time, and we found that increasing the NPC ratio led to a substantial increase in the percentage of vessels with donor cells at every time point. At the longest time point, approximately 75% of the vessels have donor cells. Increasing the ratio of NPCs does not lead to more vessels with time, but it does lead to an increase in the percentage of chimeric vessels with donor cells over time (Fig. 5e). This particularly striking in that the NPCs are only in residence for very short times.

### Soluble factor expression

While detectable NPCs are only resident in the *in vivo* microvascular network implants for short times, they impact the long-term behavioral outcomes of vessels [18]. This long-term stabilization mediated by short-term cellular signals is also evident in a 2-D coculture where BEC cords remain stable despite the withdrawal of NPCs which we investigated previously [19]. Since this stabilization is largely contact independent, soluble factors are likely to play an important role in this initial signaling. Given the importance of VEGF and BDNF in neurovascular signaling *in vivo* [59], [60] and the 2D *in vitro* model system [61], [62], ELISA assays for VEGF and BDNF were performed on the cell culture media from *in vitro* gels. To ensure reproducibility, three independent experiments were performed. Hydrogels were seeded with NPCs only, BECs only, a low ratio NPC∶BEC coculture, or a high ratio NPC∶BEC coculture. Unseeded hydrogels were used as a control. NPC only and BEC only controls were used to establish baseline expression levels.

**Figure 6:**
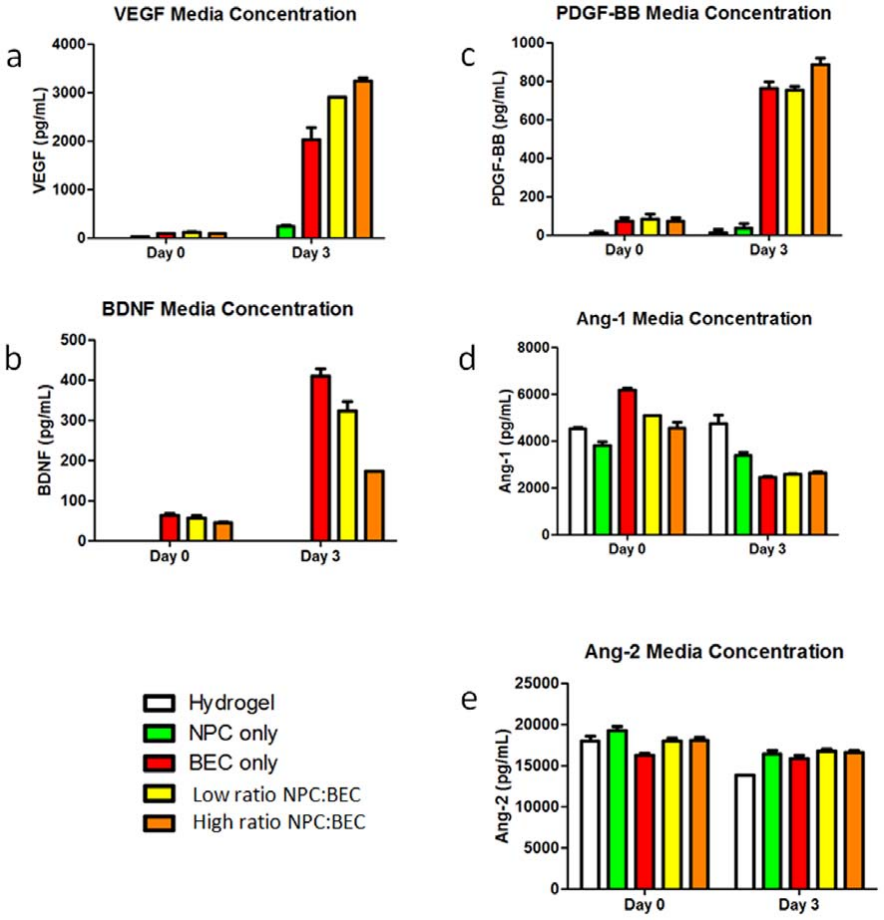
Soluble factor expression. We focused on soluble factor expression at early time points since the NPCs are in residence for only a few days post implantation. Hydrogels were seeded with no cells, NPCs only, BECs only, a low ratio NPC∶BEC coculture, or a high ratio NPC∶BEC coculture and media was sampled to determine concentrations of VEGF (a), BDNF (b), PDGF-BB (c), Ang-1 (d), and Ang-2 (e) in the pre-implantation environment. While the presence of NPCs increased media concentrations of VEGF, they decreased media concentrations of BDNF. In all cases, similar amounts of the stabilization factors PEGF-BB, Ang-1, and Ang-2 were present in the BEC only, low ratio, and high ratio cocultures at day three. Each ELISA was repeated in triplicate to ensure reproducibility. Error bars correspond to ± SE.

As in the 2D coculture experiments [19], BECs were the predominant source of VEGF and BDNF, with only a minimal amount of VEGF and no BDNF being produced by NPCs (Fig. 6a–b). Media concentrations of VEGF increased with the addition of NPCs in a dose dependent manner (fig. 6a). Conversely, media concentrations of BDNF decreased with the addition of NPCs also in a dose dependent manner. These changes in VEGF and BDNF are similar to those seen in 2D. Both NPCs and BECs increase VEGF expression when cocultured. We also know from our previous work that NPCs express more phosphorylated trkB receptors in the presence of BECs and are likely reducing the BDNF concentration.

Given the long-term effect of NPCs on the vessels and the active vascular remodeling that is present in the implants, known vessel stabilization factors, platelet-derived growth factor-BB (PDGF-BB), angiopoietin-1 (Ang-1), and angiopoietin-2 (Ang-2), may also be altered by NPCs. PDGF-BB stabilizes nascent vessels by recruiting pericytes to vascular walls. Ang-1 is a vessel maturation factor which acts to decrease the leakiness of vessels perhaps through its ability to maximize interactions of the vessel with local cells and matrix. Ang-2, on the other hand, tends to destabilize the vessel and promote its remodeling and branching. To assess the level of these factors in the pre-implantation environment, cell culture media ELISAs were performed. BECs alone produced PDGF-BB and this production was not substantially altered by the presence of NPCs (Fig. 6c). Ang-1 and Ang-2 were present to some extent in the serum containing media, but the addition of NPCs did not alter their expression levels in different treatment groups at the three day endpoint (Fig. 6d–e).

Analysis of the soluble factors suggest that the NPCs are affecting the angiogenic factors as seen previously, but not the factors associated with vessel stabilization. VEGF is an important factor for EC survival [63], which may, in part, explain the long term residence of the donor BECs, but there may be other factors we have yet to investigate that are involved. However, this model system provides a strong foundation for investigating other factors in the future.

## Discussion

In this work, we see that the ratio of donor NPCs modulates the density of donor endothelial cells with a higher ratio of NPCs leading to a greater percentage of chimeric vessels containing donor cells at all time points. The ratio of NPCs has no impact on vessel morphology or functionality and little impact on vessel density. This parallels recent work with the coculture of MSCs and BECs in collagen gels in vivo in which the cocultured cell did not substantially alter the network structure but did impact the host response [14], [15]. It is exciting that by varying the ratio of the coculture, one can modify the microvascular chimerism without substantially altering the morphology or the function of the microvascular networks. This is potentially very important in engineering microvascular networks as prevascularized templates for engineering more complex tissues.

Engineering microvessels and controlling the composition in prevascularized implants may be particularly important in systems where the host angiogenic response is compromised. Angiogenesis is compromised in elderly patients [64] where endothelial cells exhibit reduced migration [65], lower levels of angiogenic factors [66], and a decreased responsiveness to angiogenic factors that are present [67]. If we can augment the residence of donor endothelial cells we may be able to augment anastamosis and lead to better long term functional outcomes of the implants. We know from our previous work that the coculture of NPCs with endothelial cells leads to the secretion of the angiogenic signaling molecules, VEGF and BDNF, from the endothelial cells [19], and these molecules are associated with robust angiogenesis into implants [9], [68]–[70]. Augmenting donor cell residence coupled with angiogenic signaling increases the probability of a successful anastamosis between the preformed implant tubes and the host vasculature. This may overcome impaired host response in some cases.

The ability to direct the host versus donor contributions to the microvascular networks also has implications for using microvascular networks to deliver molecules systemically such as factor VIII for hemophilia [27]–[30] where the long term residence of donor cells is critical for successful outcomes. Based on the work here, we have a new platform for investigating the factors that control the chimerism and promote long term incorporation of the donor cells that secrete the factor of interest.

These findings are particularly intriguing in light of the fact that the NPCs are only resident for short times post implantation. This suggests that early signals in the implants may promote long term responses in the microvascular networks. Through the use and modulation of the NPC∶BEC system it is now possible to start to investigate what factors may be involved in the modulation of the microvascular chimerism. In our previous work, we noted that coculture of NPCs with BECs leds to greater expression of VEGF [19] and we see that here which may play a role in the early protection of the BECs. As a part of this work, we have also looked at the concentration of secreted PDGF-BB, Ang1, and Ang2, factors that are known to be important in vessel stabilization [71]–[73]. We did not see significant differences in the concentration of these secreted factors between the groups, but these are only a few of a large number of factors which can now be screened and tested in this system.

Microvascular networks are the foundation of engineering new tissues. Being able to direct and control these networks is crucial to the field. The work here provides a new method to control these networks and provides a platform to investigate the signals and mechanisms associated with the nature of these networks over time.
